# NY‐ESO‐1 antigen: A promising frontier in cancer immunotherapy

**DOI:** 10.1002/ctm2.70020

**Published:** 2024-09-14

**Authors:** Alaa Alsalloum, Julia A. Shevchenko, Sergey Sennikov

**Affiliations:** ^1^ Laboratory of Molecular Immunology Federal State Budgetary Scientific Institution Research Institute of Fundamental and Clinical Immunology Novosibirsk Russia; ^2^ Faculty of Natural Sciences Novosibirsk State University Novosibirsk Russia; ^3^ Department of Immunology V. Zelman Institute for Medicine and Psychology Novosibirsk State University Novosibirsk Russia

**Keywords:** cancer immunotherapy, cancer testis antigen, cancer vaccine, NY‐ESO‐1, tumour microenvironment

## Abstract

Significant strides have been made in identifying tumour‐associated antigens over the past decade, revealing unique epitopes crucial for targeted cancer therapy. Among these, the New York esophageal squamous cell carcinoma (NY‐ESO‐1) protein, a cancer/testis antigen, stands out. This protein is presented on the cell surface by major histocompatibility complex class I molecules and exhibits restricted expression in germline cells and various cancers, marking it as an immune‐privileged site. Remarkably, NY‐ESO‐1 serves a dual role as both a tumour‐associated antigen and its own adjuvant, implying a potential function as a damage‐associated molecular pattern. It elicits strong humoural immune responses, with specific antibody frequencies significantly correlating with disease progression. These characteristics make NY‐ESO‐1 an appealing candidate for developing effective and specific immunotherapy, particularly for advanced stages of disease. In this review, we provide a comprehensive overview of NY‐ESO‐1 as an immunogenic tumour antigen. We then explore the diverse strategies for targeting NY‐ESO‐1, including cancer vaccination with peptides, proteins, DNA, mRNA, bacterial vectors, viral vectors, dendritic cells and artificial adjuvant vector cells, while considering the benefits and drawbacks of each strategy. Additionally, we offer an in‐depth analysis of adoptive T‐cell therapies, highlighting innovative techniques such as next‐generation NY‐ESO‐1 T‐cell products and the integration with lymph node‐targeted vaccines to address challenges and enhance therapeutic efficacy. Overall, this comprehensive review sheds light on the evolving landscape of NY‐ESO‐1 targeting and its potential implications for cancer treatment, opening avenues for future tailored directions in NY‐ESO‐1‐specific immunotherapy.

**Highlights:**

- Endogenous immune response: NY‐ESO‐1 exhibited high immunogenicity, activating endogenous dendritic cells, T cells and B cells.
- NY‐ESO‐1‐based cancer vaccines: NY‐ESO‐1 vaccines using protein/peptide, RNA/DNA, microbial vectors and artificial adjuvant vector cells have shown promise in enhancing immune responses against tumours.
- NY‐ESO‐1‐specific T‐cell receptor‐engineered cells: NY‐ESO‐1‐targeted T cells, along with ongoing innovations in engineered natural killer cells and other cell therapies, have improved the efficacy of immunotherapy.

## Graphical abstract

The provided figure effectively serves as a visual summary of the therapeutic strategies targeting New York esophageal squamous cell carcinoma (NY‐ESO‐1), a cancer–testis antigen known for its high immunogenicity. It illustrates the interaction between endogenous immune cells (CD4+ T cells, CD8+ T cells, B cells and dendritic cells) and NY‐ESO‐1‐specific T‐cell receptor‐engineered cells (including natural killer cells, CD4+ T cells and CD8+ T cells) in targeting tumours. The figure also highlights various NY‐ESO‐1‐based cancer vaccine approaches, including protein/peptide‐based, RNA/DNA‐based, artificial adjuvant vector cells‐based and microbial vector‐based strategies, all of which are designed to enhance immune responses against tumours expressing NY‐ESO‐1.

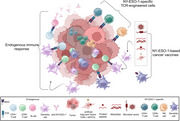

## 1 BACKGROUND

In the realm of cancer therapeutics, immunotherapy emerges as a pioneering strategy that harnesses the inherent capabilities of the immune system to combat malignancies. Diverging from traditional treatments such as chemotherapy and radiation, immunotherapy introduces a precision‐oriented approach, offering the potential to minimise systemic toxicity.^1^ As we delve into unlocking the full potential of cancer immunotherapy, a central and formidable challenge arises—identifying the optimal tumour antigens.^2^

The success of immunotherapy crucially hinges on the immune system's capacity to identify cancer cells as foreign entities, triggering a precise and potent response. However, the inherent heterogeneity of tumours poses a significant impediment, as not all cancer cells manifest readily discernible antigens amenable to immune targeting.^3^

Tumour antigens, analogous to unique molecular signatures, serve as distinctive markers that distinguish cancer cells from their healthy counterparts. The difficulty lies in precisely pinpointing these elusive antigens amid the heterogeneous nature of tumour cell populations. The emergence of novel host molecules, known as tumour‐specific antigens or tumour‐associated antigens (TAA), characterises cancer cells, facilitating the immune system's ability to recognise cancerous tissue.^4^ However, the expression of these antigens typically fails to result in the efficient eradication of tumour cells, attributed to various factors, including inconsistent antigen expression, immunological tolerance, low affinity between the T‐cell receptor (TCR) and the major histocompatibility complex (MHC)/peptide complex, as well as the presence of an immunosuppressive microenvironment.^5^

Distinct from the majority of antigens associated with tumours, a family of antigens known as cancer–testis antigens (CTA) is encoded by 276 genes, constituting over 70 gene families.^6^ These CTAs manifest notable immunogenicity, characterised by their unique expression in immune‐privileged organs—the testis and placenta, which lack MHC alleles. Furthermore, they consistently appear in tumour tissues.^7^

Within this scenario, the New York esophageal squamous cell carcinoma (NY‐ESO‐1) antigen takes center stage as a member of the CTA family. Also recognised as CTA 1B (CTAG1B), this antigen is encoded by the CTGAG1B gene situated on the Xq28 region of the X chromosome. Structurally, it is a 180‐amino acid protein weighing 18 kDa, distinguished by its immunogenicity among CTAs. It has been demonstrated to hold epitopes for humoural and cellular responses in its glycine‐rich N‐terminal region and a hydrophobic C‐terminal region with a conserved protein domain family Pcc1 (Pcc‐1).^8^, ^9^, ^10^ Although the biological functions of NY‐ESO‐1 are not extensively characterised, insights into its potential roles can be gleaned from its structural composition and expression patterns. The presence of the conserved Pcc‐1 domain has suggested a plausible involvement in regulating cell cycle progression and growth.^11^

During embryonic development, it has been shown that NY‐ESO‐1 expression initiates early in the germ cells of the testis and ovaries around 13 weeks, reaching its pinnacle between 22 and 24 weeks, followed by a rapid decrease.^12^ The decline in NY‐ESO‐1 expression during differentiation has highlighted its non‐existence in normal healthy tissues. This is crucial, as therapeutic interventions targeting NY‐ESO‐1 remain unhindered by potential implications of normal tissue damage.^13^ In the adult testis, it has been found that NY‐ESO‐1 expression persists in spermatogonia and primary spermatocytes, excluding post‐meiotic cells or testicular somatic cells.^14^, ^15^ Furthermore, there are low levels of RNA expression, but not protein expression, for NY‐ESO‐1 that have been observed in ovarian and endometrial tissue, although its biological significance remains unclear.^16^, ^17^

In cancer, it has been found that the reactivation of NY‐ESO‐1 expression is regulated by a specific epigenetic process. This intricate mechanism involves the meticulous recruitment and interaction of essential proteins, including histone deacetylases, histone methyltransferases, DNA methyltransferases and transcription factors. Mechanistically, pivotal to the regulation of NY‐ESO‐1 are multi‐protein complexes such as HDAC1‐mSin3A‐NCOR1, Dnmt3b‐HDAC1‐Egr1 and Dnmt1‐PCNA‐UHRF1‐G9a.^18^ Additionally, preclinical studies have consistently demonstrated that epigenetic drugs demethylation such as decitabine (5‐aza‐2′‐deoxycytidine, DAC) led to increased NY‐ESO‐1 expression specifically in tumour cells, which stimulated specific immune responses and resulted in reduced tumour burden and prolonged survival in several mouse models.^19^, ^20^, ^21^

NY‐ESO‐1 has been found to express in many tumours, displaying distinct expression levels among them. For example, it has been shown that myxoid and round cell liposarcoma exhibit the highest NY‐ESO‐1 expression rates (89%–100%).^22^, ^23^ Subsequently, neuroblastoma displays an (82%) expression rate, followed by synovial sarcoma (80%), melanoma (46%) and epithelial ovarian cancer (EOC, 43%).^24^, ^25^, ^26^, ^27^, ^28^, ^29^ The expression of NY‐ESO‐1 has been instrumental in distinguishing myxoid liposarcoma (MLS) from other mesenchymal tumours.^30^ Recent studies have demonstrated that NY‐ESO‐1 is highly expressed in MLS, with a sensitivity of 84.4% and specificity of 100% for MLS diagnosis. Furthermore, NY‐ESO‐1 expression has been associated with poor overall survival (OS) rates in MLS patients, highlighting its potential as both a diagnostic and prognostic marker for MLS among mesenchymal myxoid neoplasms.^31^

The robust expression of NY‐ESO‐1 in synovial sarcoma has also been proposed as a marker to differentiate synovial sarcoma from other spindle cell neoplasms, in which NY‐ESO‐1 expression is rare such as leiomyosarcoma, cellular schwannoma and dermatofibrosarcoma protuberans.^32^, ^33^

This homogeneity in NY‐ESO‐1 expression has led to the potential for these tumours to be promising candidates for immunotherapy targeting the NY‐ESO‐1 antigen. In vitro experiments have confirmed the susceptibility of MLS cell lines to NY‐ESO‐1‐specific T‐cell lysis, supporting the potential for targeted therapy.^29^ Furthermore, studies on NY‐ESO‐1‐positive ovarian cancer patients undergoing antigen‐specific immunotherapy have demonstrated enhanced responses and improved OS rates.^34^ Early clinical data have also shown promising responses in NY‐ESO‐1‐positive melanoma and synovial sarcoma treated with T cells engineered to express NY‐ESO‐1‐reactive TCRs.^35^ This underscored its potential efficacy without the severe off‐target effects observed with other CTAs, such as MAGE‐A3, which have been associated with neurotoxicity and cardiotoxicity due to cross‐reactivity with normal tissues.^36^, ^37^ These findings indicate that immune modulation in NY‐ESO‐1‐positive patients can be directed towards more favourable clinical outcomes using NY‐ESO‐1‐specific targeted therapy.

 Moreover, significantly higher expression of NY‐ESO‐1 has been observed in triple‐negative breast cancers (TNBC) compared to estrogen receptor‐positive tumours, which inherently have limited therapeutic options. This suggests that NY‐ESO‐1 could be leveraged to develop targeted immunotherapies, offering a new treatment avenue for TNBC patients.^38^ Therefore, numerous ongoing clinical trials are assessing the feasibility of NY‐ESO‐1 as a target for cancer therapy.

This review offers a comprehensive perspective on NY‐ESO‐1 as a promising target for cancer immunotherapy, emphasising its role as a highly immunogenic tumour antigen and its capacity to elicit immune responses across various malignancies. The review aims to elucidate the critical role of NY‐ESO‐1 in developing targeted immunotherapeutic strategies, drawing on insights from both clinical and preclinical trials to advance cancer treatment approaches.

### 1.1 NY‐ESO‐1 as an immunogenic tumour antigen

**FIGURE 1:**
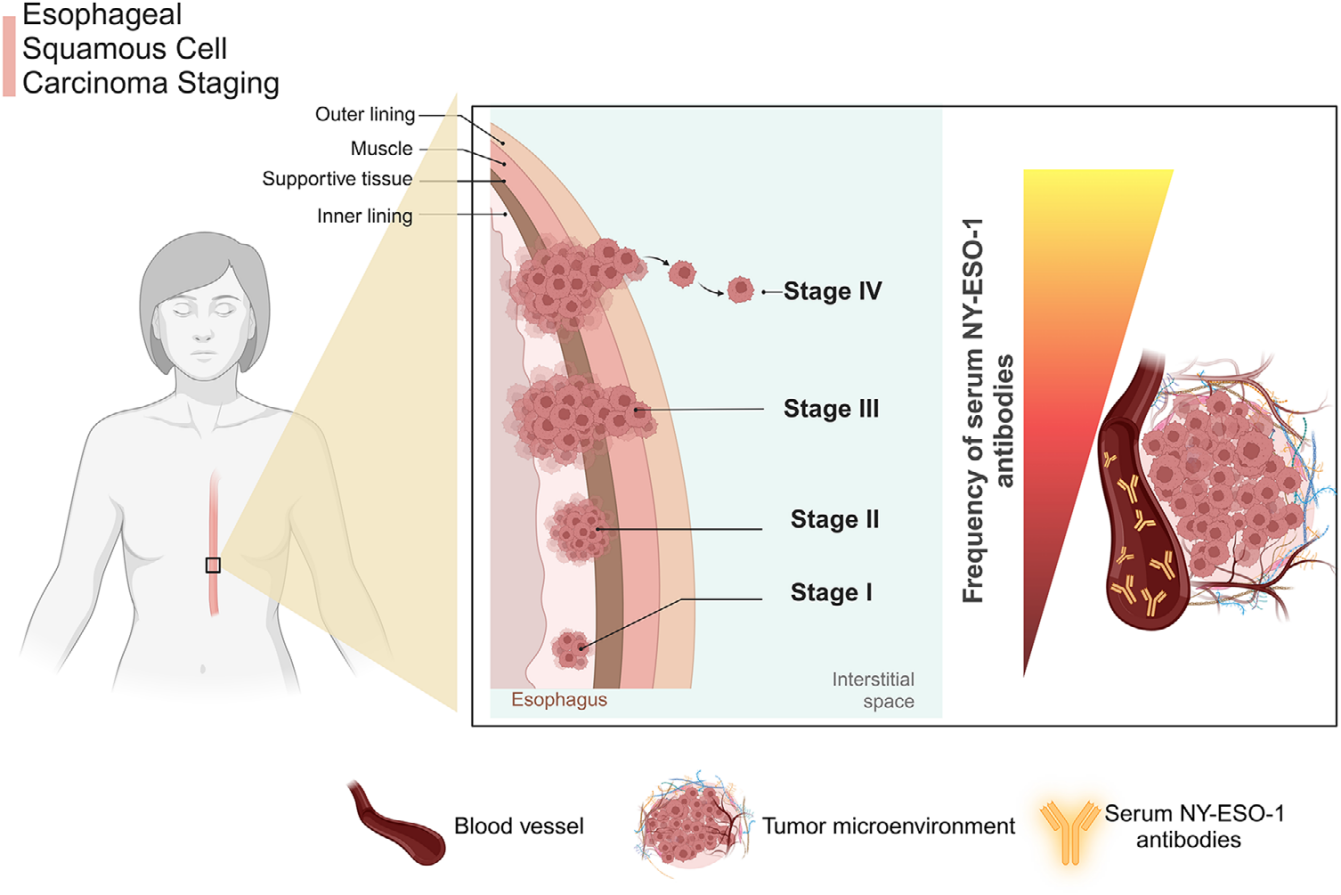
Frequency of serum New York esophageal squamous cell carcinoma (NY‐ESO‐1) antibodies in patients with esophageal carcinoma according to the tumour stage. The positive rates of patients with esophageal carcinoma gradually increased according to the tumour stage. Even in patients with stage I tumours, the positive rate was 16%, which was higher than that of the other serum marker such as carcinoembryonic antigen (CEA) and cytokeratin fragment 21‐1 (CYFRA 21‐1). It was comparable to squamous cell carcinoma antigen (SCC‐Ag) and serum p53, as reported by Oshima et al.^45^

The revelation of NY‐ESO‐1 as a tumour antigen emerged from its ability to stimulate measurable antibody responses in individuals with cancer. The initial manifestations of a spontaneous immune response against NY‐ESO‐1 was observed in a patient diagnosed with esophageal cancer.^9^ Subsequent investigations, encompassing an extensive survey of sera from 234 cancer patients aimed at establishing an ELISA‐based screening system to detect antibody responses to CTAs, underscored that NY‐ESO‐1 is highly immunogenic. It elicited spontaneous antibody responses in approximately 50% of patients with NY‐ESO‐1‐expressing tumours. In contrast, antibody responses to other CTAs, such as MAGE‐1, MAGE‐3 and SSX2, were observed at a much lower frequency.^39^ Moreover, specific antibody titres against NY‐ESO‐1 have been shown to correlate with disease progression and heightened tumour burden (Figure 1).^40^ For example, in a study of 72 patients with grade 3 transitional cell carcinoma, 12.5% (nine patients) exhibited seropositivity for NY‐ESO‐1 antibodies, all of whom had either muscle‐invasive tumours or carcinoma in situ, while no antibodies were detected in patients with grade 1 or 2 tumours.^41^ Additionally, humoural immune responses against the NY‐ESO‐1 protein were found in prostate cancer patients, but not in those with benign prostatic hyperplasia.^42^ In another study comprising 363 gastric cancer patients, NY‐ESO‐1 antibodies were found in 3.4% (6/176) of stage I, 4.4% (2/45) of stage II, 25.3% (17/67) of stage III and 20.0% (16/75) of stage IV patients. Interestingly, patients who underwent surgical intervention and did not experience subsequent relapse showed consistent decreases in NY‐ESO‐1 antibodies.^43^ Luetkens et al. investigated the immunological function and precise specificity of autologous serological responses targeting NY‐ESO‐1 in multiple myeloma patients following allogeneic stem cell transplantation. Although NY‐ESO‐1 antibodies were scarce, they exhibited the ability to activate complement, enhance CTA uptake by cells and have specific characteristics such as IgG3 restriction. This would argue for the idea that anti‐NY‐ESO‐1‐specific antibodies are in general capable of contributing to tumour control if an appropriate immune context is provided.^44^

Amidst the predominant focus on a specific cancer type in these studies, the comprehensive investigation led by Oshima et al. stands out. They have rigorously examined serological specimens from a large cohort of patients with various cancer types, and have successfully identified serum NY‐ESO‐1 antibodies (s‐NY‐ESO‐1‐Abs) as a distinctive biomarker for esophageal cancer. The positive rate for s‐NY‐ESO‐1‐Abs (32%) was notably higher in esophageal cancer patients compared to other cancer types (Table 1). It is of note that the analysis of samples from healthy individuals revealed no spontaneous NY‐ESO‐1 humoural response.^45^

**TABLE 1:** Frequency of serum New York esophageal squamous cell carcinoma (NY‐ESO‐1) antibodies in different cancer types, as documented by Oshima et al.

Cancer type	Number of cases	Serum NY‐ESO‐1 antibodies (%)
Esophageal cancer	172	55 (32.0%)
Lung cancer	269	33 (12.3%)
Hepatocellular carcinoma	91	11 (12.1%)
Gastric cancer	313	33 (10.5%)
Colorectal cancer	262	22 (8.4%)
Breast cancer	365	26 (7.1%)
Prostate cancer	358	37 (10.3%)
Normal	74	0 (.0%)

NY‐ESO‐1 has been found to trigger a cellular immune response. The initial observation of a concurrent humoural and cellular response to NY‐ESO‐1 was documented in a patient with metastatic melanoma, manifesting a robust antibody response. Three human leukocyte antigen (HLA)‐A2‐restricted epitopes within NY‐ESO‐1 were identified as CD8+ cytotoxic T lymphocyte (CTL) recognition sites, positioned between residues 155 and 167 of the NY‐ESO‐1 protein, with overlapping sequences. The peptides are at positions 155−163 (QLSLLMWIT), 157−165 (SLLMWITQC) and 157−167 (SLLMWITQCFL).^10^ In subsequent investigations, the research team discovered three HLA‐DRB40101−0103 MHC class II epitopes, recognised by CD4+ T lymphocytes in two melanoma patients.^46^ And others have identified the HLADRB1*0401‐restricted NY‐ESO‐1 119−143 peptide^47^, ^48^ and HLA‐DP4‐restricted NY‐ESO‐1 p116−135 peptide.^49^ Moreover, it was noted that the peptide harboring the HLA‐DP4‐restricted epitope has the capacity to stimulate HLA‐A2‐restricted CD8+ T cells.^50^ Other study provided a comprehensive picture of naturally occurring CD4+ T‐cell responses to NY‐ESO‐1, identifying peptide 87−98, peptide 108−119 and peptides 121−132 and 145 −156 restricted by HLA‐DP4 and HLA‐DR7.^51^ Neumann et al. further identified NY‐ESO‐1 134 −148 peptide, stimulating T‐cell responses limited to the HLA‐DRB1 subtypes *0101, *0301, *0401 and *0701.^52^

Intriguingly, among patients with advanced‐stage melanoma treated with ipilimumab, research has revealed that individuals demonstrating NY‐ESO‐1 seropositivity alongside the presence of CD8+ T cells experienced more frequent clinical benefits and enhanced survival compared to those lacking detectable CD8+ T‐cell responses.^53^ This understanding is crucial as it offers evidence that therapeutic boosting of humoural and cellular responses is a key control for NY‐ESO‐1 expressing tumours.

**FIGURE 2:**
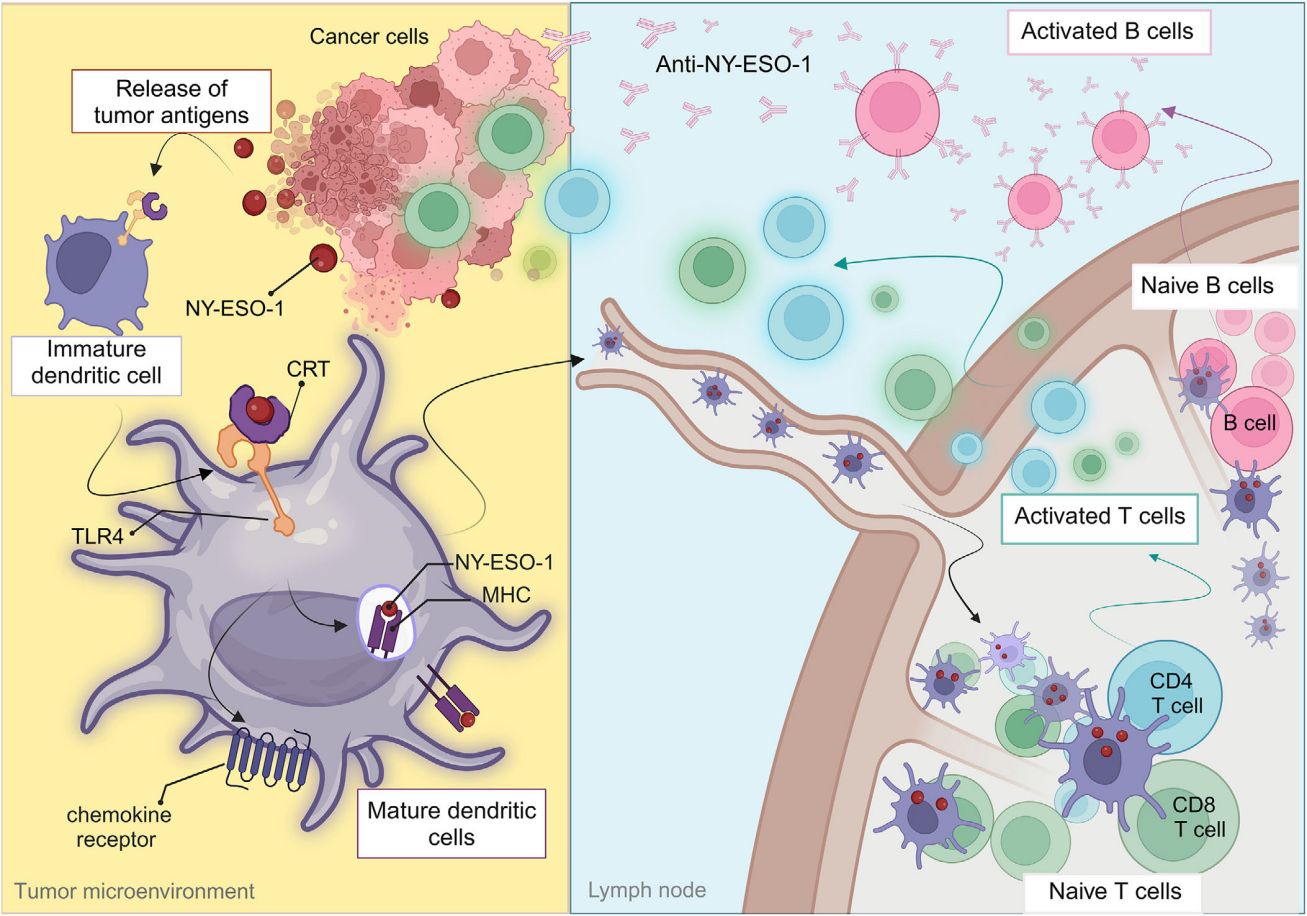
Hypothetical mechanism of New York esophageal squamous cell carcinoma (NY‐ESO‐1) immunogenicity in tumour microenvironment. NY‐ESO‐1 released from necrotic cancer cells interacts with the CRT–TLR4 complex on the surface of immature dendritic cells (DCs). The complement C1q receptor (CRT), lacking a transmembrane domain, laterally associates with Toll‐like receptor 4 (TLR4) as a co‐receptor. The binding of NY‐ESO‐1 to CRT–TLR4 induces phagocytosis, leading to the maturation of DCs. Subsequently, mature DCs migrate to the lymph nodes and present NY‐ESO‐1 peptides, initiating a cascade of immunological events that result in spontaneous immunity against various cancer types.

A subsequent study led by Zeng et al. introduced a hypothesis to elucidate the intrinsic immunogenicity of NY‐ESO‐1 in human. Within the tumour microenvironment, NY‐ESO‐1, released from necrotic cancer cells, engages with complement C1q receptor and Toll‐like receptor 4 (TLR4) on the surface of immature dendritic cells (DCs). This binding initiates phagocytosis and elicits a danger signal.^54^ Consequently, DCs undergo maturation and present NY‐ESO‐1 peptides (Figure 2). It has been postulated that NY‐ESO‐1 serves a dual role as both a TAA and an endogenous adjuvant.^55^ Despite the necessity for further elucidation concerning the precise mechanisms governing the interaction between NY‐ESO‐1 and DCs, it has been demonstrated that NY‐ESO‐1 meets the criteria for a cancer‐derived damage‐associated molecular pattern.^56^ Subsequent research has aimed to explore NY‐ESO‐1 as an adjuvant for targeting DC‐surface receptors in a mouse model. This was achieved by utilising a vaccine consisting of genetically modified Renca tumour cells expressing NY‐ESO‐1. This vaccine exhibited efficacy in reducing tumour growth compared to Renca cells expressing a control protein GFP. Additionally, the therapeutic effect correlated with heightened DC activation. Furthermore, the activation of DCs by Renca‐ESO was attenuated in TLR4^−/−^ mice, which lends support to the hypothesis.^57^

These insights served as pivotal gateways, opening avenues for the formulation of cutting‐edge strategies and vaccines to tackle NY‐ESO‐1‐positive tumours.

### 1.2 NY‐ESO‐1 cancer vaccines

Vaccines represent as an active form of immunotherapy, reinforcing the patient's immune system in its fight against cancer. The initial clinical trials for the NY‐ESO‐1 cancer vaccine, conducted over a decade ago, led to significant progress in the discovery of peptides and the formulation of vaccines. This encompasses a diverse spectrum of modalities, including peptides, proteins, DNA, mRNA, viral vectors, bacterial vectors, artificial adjuvant vector cells (aAVCs) and DC‐based vaccines.

## 2 PEPTIDE AND PROTEIN‐BASED VACCINES

**FIGURE 3:**
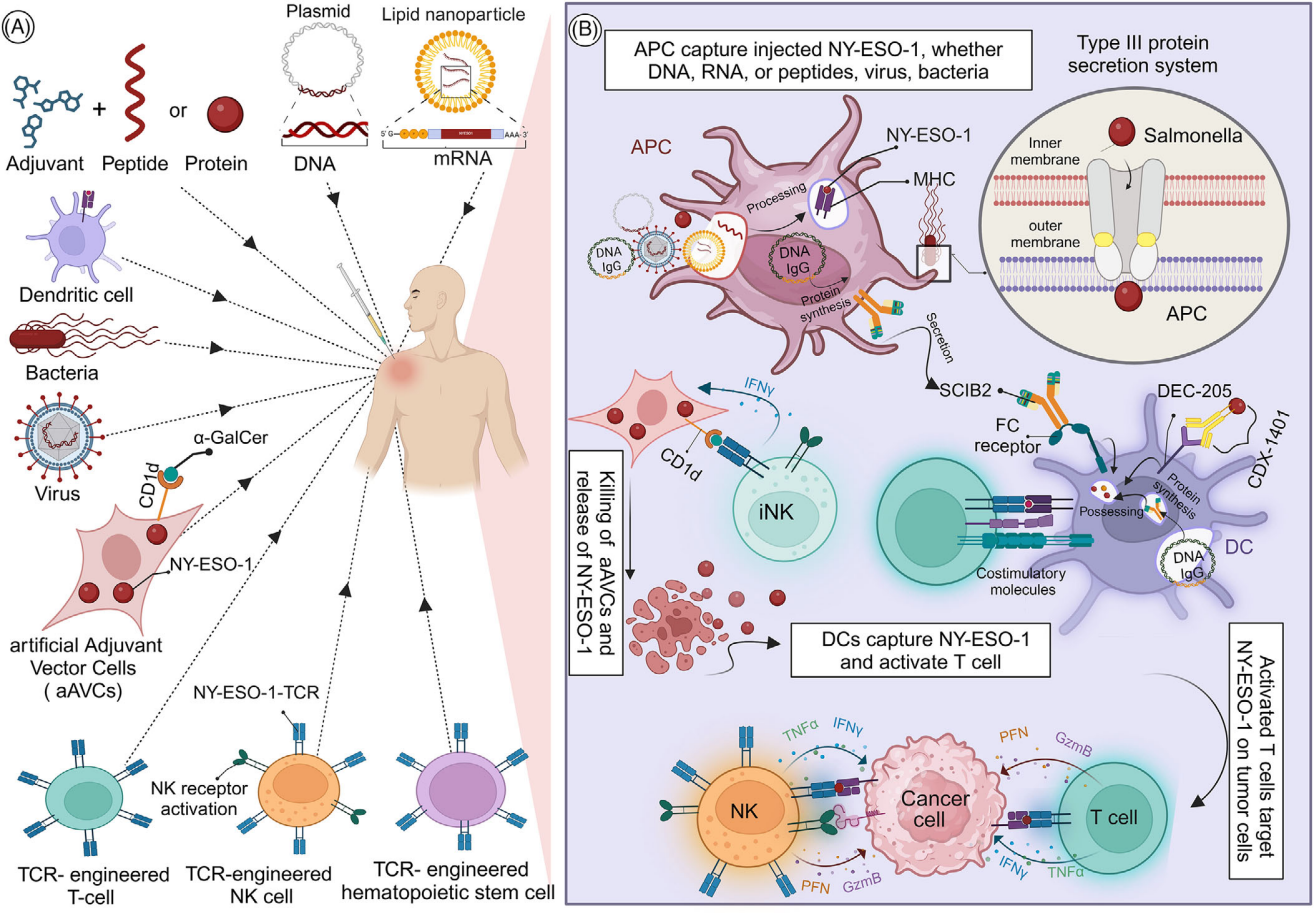
Schematic overview of New York esophageal squamous cell carcinoma (NY‐ESO‐1) immunotherapy in cancer: (A) strategies and (B) mechanisms. (A) These include peptides, proteins, DNA, mRNA, dendritic cells, viruses, bacteria, artificial adjuvant vector cells (aAVCs), and T‐cell receptor (TCR) engineered adoptive cell‐based vaccines. (B) APCs capture the injected NY‐ESO‐1, whether in the form of DNA, RNA, peptides, or viruses, and present it to the immune system, resulting in the activation of specialized T‐cells targeting NY‐ESO‐1 on tumor cells. The DNA IgG form can either be internalized, processed by APCs at the injection site, leading to its secretion as the protein form of IgG, such as SCIB2. SCIB2 then binds to the high‐affinity Fc receptor on dendritic cells, triggering cross‐presentation, or it can be directly presented by dendritic cells themselves. In another approach, CDX‐1401 is an infusion protein comprising NY‐ESO‐1 tumor protein and a human monoclonal antibody targeting the dendritic cell receptor DEC‐205, thus facilitating further antigen presentation. Additionally, aAVCs present GalCer on CD1d molecules, activating invariant natural killer T (iNKT) cells to recognize and eliminate the aAVCs and subsequently releasing tumor‐associated antigens. Dendritic cells (DC) then capture and present these antigens to the immune system. Modified Salmonella typhimurium delivers the NY‐ESO‐1 antigen directly into antigen‐presenting cells using a type III protein secretion system, facilitating its transport across cellular membranes and into the cytoplasm of the antigen‐presenting cells. T‐cell receptor (TCR) engineered cells are tailored to precisely target NY‐ESO‐1 on tumor cells. It is noteworthy that the engineered NK cells retain their complete set of native receptors and induce cytotoxic effects through mechanisms extending beyond TCR specificity.

Protein and peptide vaccines are designed to mimic specific TAAs expressed by cancer cells, employing purified, recombinant or synthetically engineered epitopes and proteins. Within the tumour microenvironment, antigen‐presenting cells (APCs), such as DCs, process and present these peptides bound to MHC molecules. The recognition of these peptide–MHC complexes by CD8+ CTLs triggers their activation, leading to the expansion of tumour‐specific CTLs capable of targeting and eliminating cancer cells expressing TAAs (Figure 3).^58^

Numerous clinical trials exploring NY‐ESO‐1 protein or peptide‐based vaccines have emphasised the co‐administration of recombinant peptides/proteins alongside adjuvants. This approach aims to bolster chemical stability and enhance immune‐stimulating properties, thus facilitating the generation of a robust immune response.^59^, ^60^, ^61^, ^62^, ^63^, ^64^, ^65^, ^66^, ^67^, ^68^, ^69^, ^70^, ^71^

In a pivotal phase I trial (NCT00616941, Table 2), researchers assessed the impact of polyinosinic:polycytidylic acid (poly‐ICLC) and Montanide on NY‐ESO‐1 vaccination for ovarian cancer, utilising overlapping long peptides (OLPs) from NY‐ESO‐1. Three cohorts were established: cohort 1 received OLP alone for safety assessment, cohort 2 received Montanide‐emulsified OLP, while cohort 3 incorporated poly‐ICLC, which is known for its TLR ligands, into the emulsion. OLP alone failed to elicit any measurable immune responses, whereas Montanide induced NY‐ESO‐1‐specific CD4+ T cells with inconsistent antibody and CD8+ T‐cell responses. The incorporation of poly‐ICLC into the emulsion facilitated the generation of NY‐ESO‐1‐specific antibodies, enhanced CD4+ T‐cell responses, and sustained CD8+ T‐cell responses.^72^

**TABLE 2:** An overview of New York esophageal squamous cell carcinoma (NY‐ESO‐1)‐related therapies: key findings from clinical trials.

Identifier	Phase	Type of tumour	Formulation of therapy	Epitope	Results
NCT00616941^72^	I	EOC	Peptide vaccine with Montanide and/or poly‐ICLC	Synthetic OLP from a human tumour self‐antigen: NY‐ESO‐1:79‐108; NY‐ESO‐1:100‐129; NY‐ESO‐1:121‐150; NY‐ESO‐1:142‐173	Safe administration of NY‐ESO‐1 OLP vaccine. Rapid induction of integrated immune responses (antibody, CD8+, CD4+) in nearly all patients
NCT02129075^77^	II	Melanoma	CDX‐1401: DEC‐205/NY‐ESO‐1 fusion protein vaccine in combination with poly‐ICLC; CDX‐301	NY‐ESO‐1:157−165	ced innate immune cells (DCs, monocytes and NK cells) Increased anti‐NY‐ESO‐1 antibodies and NY‐ESO‐1‐specific T cells in the cohort pre‐treated with CDX‐301 (Flt3 ligand)
NCT00948961^76^	I	Melanoma, ovarian, sarcoma, non‐small cell lung, colorectal cancers	CDX‐1401 in combination with resiquimod and/or poly‐ICLC	NY‐ESO‐1:157−165	No dose‐limiting or grade 3 toxicities Stable disease: 13 patients, with a median duration of 6.7 months 6/8 patients experienced objective tumour regression (who received immune checkpoint inhibitors within 3 months after CDX‐1401) Two patients showed tumour regression (∼20% shrinkage in target lesions)
NCT02410733^86^	I	Melanoma	BNT111: a FixVac RNA vaccine, containing four tumour‐associated antigens, including full‐length sequences of NY‐ESO‐1, Tyrosinase, MAGE‐A3 and TPTE		BNT111 generated antigen‐specific CD4+ and CD8+ T cells Most patients showed PR BNT111‐induced T cells effectively targeted melanoma cells with strong cytotoxicity T‐cell responses persisted for more than 1 year Two melanoma patients achieved >35% tumour regression with BNT111 in combination with anti‐PD1 therapy
LUD00‐014^96^	II	Melanoma	Recombinant vaccinia‐NY‐ESO‐1 (rVNY‐ESO‐1) and recombinant FowlpoxNY‐ESO‐1 (rF‐NY‐ESO‐1)		Among 25 melanoma patients: CBR: 72%. ORR: 14% Median PFS: 9 months Median OS: 48 months. Robust T‐cell responses and effective antibody production against NY‐ESO‐1 in the majority of patient Patients who had preexisting NY‐ESO‐1‐specific immunity experienced substantial benefits CD8+ T‐cell responses predominantly targeted NY‐ESO‐1 epitopes in regions p81‐110 and p151‐170, with lower responses in p119‐143 regions
LUD02‐012^96^	II	EOC			Among 22 EOC patients: Median PFS: 21 months Median OS: 48 months Strong T‐cell responses and effective antibody production in the majority of patients CD8+ T‐cell responses predominantly targeted NY‐ESO‐1 epitopes in the p81‐110 region, with lower responses in the p119‐160 regions of NY‐ESO‐1
NCT02122861^99^	Case report	Synovial sarcoma	LV305: lentiviral vector vaccine targeting DC, from the ZVex platform, encoding antigen NY‐ESO‐1	The full‐length protein NY‐ESO‐1	Well‐tolerated treatment with no adverse events above grade 2 Achieved over 85% regression in synovial sarcoma following three injections of LV305 Sustained robust NY‐ESO‐1‐specific CD4+ and CD8 T‐cell response observed for over 2.5 years LV305‐triggered long‐lasting antitumour effector‐memory T‐cell responses
NCT02609984^101^	II	Synovial sarcoma myxoid liposarcoma	CMB305: LV305, a NY‐ESO‐1‐expressing lentiviral vector, and G305, a recombinant adjuvanted NY‐ESO‐1 protein; atezolizumab		CMB305 plus atezolizumab led to increased NY‐ESO‐1‐specific T cell and antibody responses compared to atezolizumab alone
NCT02692976^105^	II	Prostate cancer	Blood‐derived mDCs and pDCs stimulated with protamine/mRNA and loaded with NY‐ESO‐1, MAGE‐C2 and MUC		Both DC subset vaccines induced NY‐ESO‐1‐specific T cells in 71% of patients T‐cell presence correlated with better outcomes, median duration 27.2 months
NCT00670748^109^	II	Synovial cell sarcoma melanoma	Autologous T cells transduced with an NY‐ESO‐1‐reactive TCR; ALVAC NY‐ESO‐1 vaccine; cyclophosphamid; fludarabine	1G4‐α95:LY TCR NY‐ESO‐1:157−165 in complex with HLA‐A*0201	Among 18 patients synovial cell sarcoma: OCR: 61% 3‐year OSR: 38% 5‐year OSR: 14% Among 20 patients melanoma: OCR: 55% 3‐year OSR: 33% 5‐year OSR: 33%
NCT03250325^110^, ^111^	I/II	Synovial sarcoma	TBI‐1301: lymphocytes transduced with the retroviral vector MS3II‐TCR NY‐ESO‐1‐siTCR (siTCR‐ interfering RNA constructs that specifically downregulate endogenous TCR); cyclophosphamide	G50A:A51E TCR NY‐ESO‐1:157−165 in complex with HLA‐A*02:01/*02:06	Among the eight patients: ORR: 50.0% Median OS: 650 days CRS: 50.0% Grade 1 CRS: one patient Grade 2 CRS: three patients Adverse drug reactions: 87.5% All cases recovered with prespecified treatment
JMA‐IIA00346^119^		Advanced soft tissue sarcoma	TBI‐1301 plus CHP:NE1 (a complex of pullulan nanogel carrying an NY‐ESO‐1 with a K3 CpG oligoDNA adjuvant)		Among the three patients: One patient demonstrated tumour shrinkage that persisted for over 2 years and the sustained presence of TCR T cells One patient increased size of the target lesions before TBI‐1301 infusion and then decreased after infusion Two CRS cases: grade 1, 2 Patients with low expression NY‐ESO‐1 showed poorer response
NCT01343043^112^	I	Synovial Sarcoma	NY‐ESO‐1 SPEAR T cells: autologous T cells transduced with an NY‐ESO‐1‐reactive TCR; fludarabine; cyclophosphamide	NY‐ESO‐1/LAGE‐1‐derived SLLMWITQC peptide in complex with HLA‐A*0201 (NY‐ESO‐1c259)	Among 12 synioval scarcoma patients: ORR: 50% CR: one patient PR: five patients PFS: 15 weeks OS: 120 weeks CRS: five patients with median duration 10 days Among seven patients: Persisting of circulating NY‐ESO‐1c259TCR: 200 days Subsets of persistent T‐cell clonotypes: TSCM and TCM
NCT01352286^114^, ^115^	I/II	Multiple myeloma	NY‐ESO‐1 SPEAR T cells administered post‐autologous stem cell transplantation		Among the 25 patients: ORR: 44% PFS: 52% Very good PR: eight patients PR: one patient Stringent CR: one patient CR: one patient PFS with 13.5 months: three patients Median OS: 35.1 months
NCT02775292^116^	I	Undifferentiated pleomorphic sarcoma	NY‐ESO‐1‐reactive TCR retroviral vector transduced autologous PBL; peptide‐pulsed autologous DC vaccine; nivolumab; aldesleukin; cyclophosphamide; fludarabine phosphate	HLA‐A*02:01‐restricted NY‐ESO‐1:157−165	Sarcoma resistance to treatment may be driven by a new mechanism: loss of NY‐ESO‐1 expression due to extensive methylation of its promoter region
NCT04526899	II	Melanoma	BNT111 + cemiplimab (anti‐PD‐1)		Active, not recruiting
NCT03970746	I/II	Non‐small cell lung cancer	PDC*lung01: irradiated pDC, loaded separately with seven peptide NY‐ESO‐1, MAGE‐A3, MAGE‐A4, multi‐MAGE, SURVIVN, MUC1, and Melan‐A anti‐PD‐1		Active, not recruiting
NCT02650986	I/IIa	Advanced malignancies	NY‐ESO‐1 TCR/TGFbDNRII: autologous tumour infiltrating lymphocytes; cyclophosphamide; decitabine	HLA‐A*0201‐restricted NY‐ESO‐1	Active, not recruiting
NCT03691376	I	Ovarian, fallopian tube, or primary peritoneal cancer	NY‐ESO‐1 CD4+ TCR‐engineered HSC; autologous NY‐ESO‐1 CD8+ TCR‐engineered T cells; melphalan; aldesleukin	HLA‐A*02.1 and HLA‐DP*04‐restricted NY‐ESO‐1	Active, not recruiting
NCT06066359	I/II	Multiple myeloma	NY‐ESO‐1 TCR/IL‐15 NK cell; fludarabine phosphate; cyclophosphamide	HLA‐A*02:01‐restricted NY‐ESO‐1	Recruiting
NCT01946373	I	Melanoma	DC loaded with autologous tumour lysate and a synthetically produced peptide derived from the tumour‐associated antigen NY‐ESO‐1; autologous TIL; cyclophosphamide; fludarabine interleukin‐2		Recruiting
NCT01697527	II	Malignant neoplasm	NY‐ESO‐1‐reactive TCR retroviral vector transduced autologous PBL; DC vaccine; Aldesleukin; Fludarabine phosphate, cyclophosphamide; fludeoxyglucose F 18	HLA‐A*0201‐restricted NY‐ESO‐1157‐165	Active, not recruiting

Abbreviations: CBR, clinical benefit rate; CR, complete response; CRS, cytokine release syndrome; DC, dendritic cell; EOC, epithelial ovarian cancer; HLA, human leukocyte antigen; HSC, haematopoietic stem cell; mDC, myeloid dendritic cell; NK, natural killer; OCR, objective clinical responses; OLP, overlapping long peptides; ORR, overall response rate; OS, overall survival; OSR, overall survival rate; PD‐1, programmed cell death protein 1; pDC, plasmacytoid dendritic cell; PFS, progression‐free survival; poly‐ICLC, polyinosinic:polycytidylic acid; PR, partial response; TCM, central memory T cell; TCR, T‐cell receptor; TIL, tumour infiltrating lymphocyte; TSCM, T memory stem cells.

In a separate investigation, researchers analysed the CD8+ T‐cell response to a NY‐ESO‐1 peptide vaccine comprising two previously defined peptides (157–165 and 157−167), administered in conjunction with granulocyte‐macrophage colony‐stimulating factor (GM‐CSF) as a systemic adjuvant. The study demonstrated that while the CD8+ T‐cell response to the NY‐ESO‐1 peptide vaccine‐targeted multiple epitopes, only a small fraction of the induced CD8+ T cells recognised the naturally processed target on tumour cells.^73^ Consequently, although the CD8+ T‐cell response to the NY‐ESO‐1 peptide vaccine yielded a broad, multi‐epitope‐directed response, its efficacy in precisely targeting tumours was limited. This prompted the question of whether the induced immune response mirrors or complements the natural immune response against endogenously expressed antigens.

Researchers conducted a comprehensive analysis of the structural intricacies of this phenomenon, investigating the TCR characteristics of both naturally occurring and vaccine‐induced NY‐ESO‐1‐specific CTLs. Their investigations revealed that CTLs from both categories exhibited conserved yet distinct TCR features. It is important to note that the TCR repertoire elicited by synthetic peptide vaccination may not accurately reflect the naturally processed antigen.^74^, ^75^ Given the intricate nature of the CD8+ T‐cell repertoire induced by synthetic peptide vaccination, it is of the utmost importance to meticulously delineate the targeted epitope and the corresponding peptide to serve as an immunogen. This is necessary to ensure precise tumour targeting.

In addition, numerous clinical trials have combined NY‐ESO‐1 vaccination with other modalities to augment the efficacy of the therapy. For instance, researchers employed an infusion protein NY‐ESO‐1 tumour protein along with a human monoclonal antibody targeting the DC receptor DEC‐205 (CDX‐1401) (NCT00948961, Table 2, Figure 3B).^76^ In in a phase II study (NCT02129075, Table 2), CDX‐1401 was administered to patients with resected melanoma following pretreatment with CDX‐301, a recombinant human Flt3 ligand (Flt3L), which is known to expand circulating DCs, and poly‐ICLC (a TLR3 agonist). Bhardwaj et al. reported that CDX‐301 pretreatment led to increased DC populations and enhanced responses to NY‐ESO‐1 elicited by CDX‐1401 vaccination. These findings indicate the potential of this regimen as a prophylactic vaccine against disease recurrence in high‐risk melanoma patients.^77^

Another investigation utilised a novel antigen delivery system called Cholesteryl pullulan, which presents multiple epitope peptides to the MHC class I and II pathways. They conducted a first‐in‐human clinical trial of the NY‐ESO‐1 protein cancer vaccine alongside the adjuvant MIS416 in patients with NY‐ESO‐1‐expressing refractory solid tumours. The results demonstrated that the therapy was safe and well tolerated, with manageable adverse events. However, there was no significant increase in the immune response observed with escalating doses of MIS416. Additionally, the study referenced a preclinical investigation that exhibited promising tumour suppression when the vaccine was combined with an anti‐programmed cell death protein 1 (PD‐1) monoclonal antibody, hinting at a potential future direction in therapy.^67^

However, achieving precise elimination of regulatory T cells (Tregs) in patients poses a significant challenge. Although low‐dose cyclophosphamide has demonstrated effectiveness in enhancing antigen‐specific CD4+ T‐cell responses to the NY‐ESO‐1/ISCOMATRIX vaccine among individuals with advanced melanoma compared to vaccination alone, it has not yielded notable reductions in Tregs.^78^ The lack of a defined hierarchy in immunosuppression and the dynamic nature of suppressive mechanisms within solid tumours remains a formidable obstacle in the context of vaccine therapy.^79^ Consequently, several clinical trials have been initiated to explore combination therapies involving peptide NY‐ESO‐1‐based cancer vaccines, however, to date, no publications have emerged from these studies. These trials have focused on evaluating the efficacy of various approaches, including checkpoint inhibitors (e.g., NCT01176474, NCT02737787, NCT01176461), demethylating agents (e.g., NCT02750995, NCT03358719, NCT01673217) and an indoleamine 2,3‐dioxygenase inhibitor (NCT02166905) across various types of cancer.

As efforts to enhance peptide and protein vaccines progress, the persistent challenges in achieving consistent and robust immune responses have driven the exploration of new strategies, including nucleic acid‐based vaccines.

## 3 NUCLEIC ACID‐BASED VACCINES

Nucleic acid‐based vaccines represent a significant advancement in immunotherapy, characterised by their strong adjuvant properties.^80^ These vaccines, utilising DNA or RNA, employ the genetic sequences of full‐length tumour antigens, allowing for the presentation of multiple epitopes and eliciting a robust T‐cell response (Figure 3A). In the development of RNA vaccines, RNA is synthesised in vitro from a DNA template encoding the antigen, utilising bacteriophage RNA polymerase.^81^

### 3.1 DNA vaccines

DNA vaccines utilise plasmids as vectors to transport genes encoding tumour antigens, thereby stimulating or amplifying antigen‐specific immune reactions. For instance, the NY‐ESO‐1 DNA vaccine, has been shown to elicit strong CD4+ and CD8+ T‐cell responses in cancer patients (Figure 3B). The vaccine was administered via particle‐mediated epidermal delivery (gene gun), which has demonstrated enhanced transgene expression. Nevertheless, despite the induction of antigen‐specific T‐cell responses, the clinical results primarily featured disease progression, suggesting the potential involvement of regulatory T‐cell mechanisms that might undermine the vaccine's efficacy.^82^

In addition, an intriguing strategy involves modifying existing human IgG DNA to produce tailored antibodies, which has been shown to evoke robust antitumour CD8+ T‐cell responses with high avidity, surpassing other vaccination approaches such as peptide vaccines or DNA vaccines containing epitopes within native antigens. The key feature of this approach lies in its capacity to trigger both direct and cross‐presentation of epitopes to T cells. DCs utilise various pathways for antigen processing, but the generation of the most potent T‐cell responses, characterised by high avidity, occurs when multiple pathways are employed to present the same epitope (Figure 3B).^83^

To enhance the versatility of this vaccination strategy, scientists incorporated 16 NY‐ESO‐1 epitopes, spanning over 80% of HLA phenotypes into the complementarity‐determining regions (CDRs) to develop human igg DNA (SCIB2). Engineered to target DCs in vivo via the high‐affinity Fc receptor, SCIB2 vaccination led to significant tumour regression and induced more frequent and high‐avidity potent T‐cell responses than peptide vaccination (Figure 3B). Moreover, when coupled with Treg depletion, cytotoxic T‐lymphocyte antigen‐4 (CTLA‐4) blockade, or PD‐1 blockade, SCIB2 vaccination exhibited improved long‐term survival rates in individuals with established tumours.^84^

Nonetheless, the concern regarding the potential risk of plasmid DNA integration into the host genome persist.^85^ Consequently, researchers are shifting their focus towards on exploring the potential of NY‐ESO‐1 mRNA cancer vaccines.

### 3.2 mRNA vaccines

RNA‐based cancer vaccines utilise liposomes and protamine‐mRNA nanoparticles as delivery systems, encapsulating and protecting the mRNA from degradation while facilitating its delivery into cells (Figure 3A).

In a phase I trial (NCT02410733, Table 2) for advanced melanoma patients, the FixVac (BNT111) RNA vaccine, containing four TAAs including NY‐ESO‐1, showed promising results. The vaccine induced enduring objective responses, robust T‐cell immunity, and significant antitumour effects, either alone or combined with checkpoint inhibitors (Figure 3B).^86^ These findings led to the commencement of a randomised phase II clinical trial (NCT04526899).

In another phase Ib clinical trial (NCT03164772), an mRNA‐based vaccine BI1361849 (CV9202), incorporating cancer antigens such as NY‐ESO‐1 and complexed with the cationic protein protamine for self‐adjuvating, was investigated alongside local radiation therapy in 26 stage IV non‐small cell lung cancer (NSCLC) patients. Immunomonitoring revealed heightened antigen‐specific immune responses in most patients, including increased antibody levels and functional T cells. Remarkably, one patient demonstrated a partial response, while 46.2% achieved stable disease.^87^ This study underscores the significance of refining delivery systems and adjuvants in vaccine research. Microbial vector vaccines are emerging as a promising approach, utilising microbial characteristics to act as potent adjuvants and further enhance immune responses.

## 4 MICROBIAL VECTOR VACCINES

Microbial vector vaccines are recognised for their ability to induce antigen presentation through both MHC class I and class II pathways. These vaccines harness the innate immune surveillance mechanisms of the host, steering the immune system towards targeting and eradicating tumours.

### 4.1 Bacterial vaccines

Bacteria act as intrinsic immune stimulants, harbouring pathogen‐associated molecular patterns that trigger pattern recognition receptors such as TLRs on immune cells. This engagement prompts a robust innate immune response, effectively disrupting immunological tolerance. Therefore, non‐pathogenic recombinant bacterial vectors have garnered interest for their potential application as a platform to deliver antigens in cancer vaccine development.^88^

Numerous preclinical and clinical trials have explored the efficacy of bacterial vaccines targeting NY‐ESO‐1. One such vaccine, utilising *Salmonella typhimurium*, was engineered to deliver NY‐ESO‐1 antigen directly into APCs via a type III protein secretion system (Figure 3B). This vaccine demonstrated effectiveness in mice and was capable of eliciting NY‐ESO‐1‐specific CD8+ and CD4+ T‐cell responses in cancer patients with pre‐existing NY‐ESO‐1 immunity.^89^ Follow‐up investigations demonstrated that the *S. typhimurium*‐NY‐ESO‐1 vaccine prompted the development of CD4+ T helper 1 (Th1) cells in melanoma patients without inherent NY‐ESO‐1 immunity. Notably, these Th1 cells displayed elevated glucocorticoid‐induced TNFR‐related protein (GITR) expression compared to those induced by peptide vaccines and were resistance to suppression by CD4+ CD25+ regulatory T cells in a GITR‐dependent manner.^90^

The investigation into novel mechanisms lays the groundwork for the development of multifaceted bacterial vaccines. In a phase I clinical trial (NCT01967758), a live‐attenuated *Listeria monocytogenes* vaccine (ADU‐623) engineered to express NY‐ESO‐1 and EGFRvIII were evaluated for glioma therapy. Although efficacy data are still awaited, ongoing advancements in engineered bacterial immunotherapy for cancer within clinical trials offer promising avenues for improving clinical outcomes.^91^

### 4.2 Viral vaccines

Viral‐based vaccines utilise genetically modified viruses as carriers to target cancer cells, facilitating the expression of antigens within the cells and triggering a strong CTL response, ultimately resulting in the eradication of virus‐infected cells (Figure 3).^92^

Adenovirus and vaccinia virus are among the extensively researched vectors due to their notable immune‐stimulating capabilities, especially in activating CTLs, without requiring the use of adjuvants.^93^

Most viral vectors are engineered to be replication defective to maintain a high level of biological safety. However, viral‐based cancer vaccines encounter challenges due to pre‐existing immunity against the virus used, such as vaccinia virus, a member of the poxvirus family traditionally employed in the smallpox vaccine. To address this issue, prime‐boost regimens are often implemented.

In a clinical trial involving patients with various advanced solid tumours, researchers utilised recombinant vaccinia‐NY‐ESO‐1 (rV‐NY‐ESO‐1) as the prime, followed by recombinant fowlpox‐NY‐ESO‐1 (rF‐NY‐ESO‐1) as the booster. This sequential administration aimed to bolster immune responses without eliciting host‐neutralising immunity.^94^ The prime‐boost strategy proved safe and effectively stimulated both humoural and cellular immune responses against a wide array of NY‐ESO‐1 epitopes. Among the nine patients with advanced stage III/IV melanoma, seven survived for 17 months to over 5 years (63+ months). Importantly, clonal analysis of vaccine‐induced CD8+ T cells demonstrated robust recognition of naturally processed NY‐ESO‐1 in tumour cell lines.^95^ Two parallel phase II clinical trials evaluated the efficacy of the same vaccine regimen in melanoma and EOC patients. In melanoma, the objective response rate was 14%, with a clinical benefit rate of 72%, while EOC patients showed a median progression‐free survival (PFS) of 21 months. Vaccinated individuals also exhibited strong T‐cell responses and antibody production against NY‐ESO‐1 (LUD00‐014, LUD02‐012, Table 2).^96^

In the field of cancer immunotherapy, the harnessing of T‐cell costimulation has received considerable attention as a means to enhance the immune system's ability to eliminate tumour cells. Preclinical studies with the vCP2292 vaccine [ALVAC(2) (2)‐NY‐ESO‐1(M)‐TRICOM] have shown that the integration of transgenes encoding various costimulatory molecules (TRICOM, B7‐1, ICAM‐1 and lymphocyte‐function antigen 3 [LFA‐3]) into recombinant ALVAC(2) poxviruses, together with the NY‐ESO‐1 transgene, enhances NY‐ESO‐1‐specific T‐cell immune responses.^97^

Subsequent clinical trials have investigated the combination of this vaccine with the mammalian target of rapamycin inhibitor sirolimus (NCT01536054) or GM‐CSF sargramostim in patients with various cancers (NCT00803569). However, comprehensive publications from these trials have not been provided.

Various viral vectors are currently under investigation for cancer treatment, either alone or in combination with other therapies. Albershardt et al. engineered an integration‐deficient lentiviral vector, LV305, to deliver the tumour antigen NY‐ESO‐1 to DCs through its pseudotyped envelope glycoprotein derived from the alpha virus Sindbis, which recognises the human dendritic cell‐specific intercellular adhesion molecule‐3‐grabbing non‐integrin (DC‐SIGN) (CD209) receptor on the surface of immature human DCs.^98^ A case report from a phase I clinical (NCT02122861, Table 2 (trial highlighted significant disease regression exceeding 85% in a patient with synovial sarcoma after receiving three intradermal injections of LV305, accompanied by a robust NY‐ESO‐1‐specific CD4+ and CD8+ T‐cell response that persisted for over 2.5 years post‐therapy. The treatment was well tolerated with no adverse events above grade 2, marking a significant milestone as the first successful application of lentiviral vector‐expressing NY‐ESO‐1 in human subjects, offering promising clinical and immunologic benefits for advanced‐stage cancer patients.^99^ LV305 was then used in a prime‐boost vaccine regimen, CMB305, in patients with advanced solid tumours (NCT02387125). CMB305 includes LV305 as the priming agent and G305—full‐length NY‐ESO‐1 protein with glucopyranosyl lipid A as an adjuvant—as the booster. This regimen resulted in a median OS of 26.2 months and elicited anti‐NY‐ESO‐1 antibody in 62.9% and T‐cell responses in 47.4% of patients.^100^ A phase II trial (NCT02609984, Table 2) compared the combination of CMB305 and atezolizumab (anti‐PD‐1) to atezolizumab alone in 89 patients with NY‐ESO‐1‐expressing synovial sarcoma or MLS. While the combination did not significantly improve PFS or OS, with a median OS of 18 months in both arms, it induced higher rates of NY‐ESO‐1‐specific T cells and antibody responses. Notably, patients who developed an anti‐NY‐ESO‐1 T‐cell response had longer OS.

These findings suggest that prime‐boost immunotherapies such as CMB305 could enhance NY‐ESO‐1‐engineered T‐cell responses and warrant further exploration in combination with checkpoint inhibitor.^101^

Another vaccine employing Chimpanzee Adenovirus Oxford 1 (ChAdOx1‐MAGEA3‐NY‐ESO‐1) is underway to evaluate along with chemotherapy and an immune checkpoint inhibitor for patients with NSCLC in phase I/IIa trial (NCT04908111).

In contrast to viral vector‐based strategies targeting DCs in vivo, a more direct approach involves administering DCs as an adjuvant therapy, enabling precise delivery of APCs to enhance immune responses.

## 5 DENDRITIC CELL‐BASED VACCINES

DCs, recognised as the most potent APCs, play a critical role in initiating adaptive immune responses. In this strategy, DCs are either pulsed with the peptide or transduced with the peptide, serving as both adjuvant and delivery system for vaccination. This innovative approach aims to introduce educated DCs that present specific tumour antigens to the immune system, thereby activating immune cells, particularly T cells, to target and eliminate cancerous cells (Figure 3).^102^

Numerous early clinical trials, registered on clinical.gov with identifiers, such as NCT00313508, NCT01241162, NCT01159288, NCT02775292, NCT02332889, NCT02224599 and NCT02070406, have investigated the potential of peptide‐pulsed DCs as cellular vaccines. These trials have explored the efficacy of this treatment approach both as standalone treatments and in combination with TLR3 ligands such as poly‐ICLC (NCT02334735), as well as with other immunotherapy strategies. However, the results of these trials have not yet been published.

Another aspect to consider in DC vaccine manufacturing is the transcriptional and functional variability among DC subtypes, which is influenced by their developmental origin and tissue localisation. In the bloodstream, two main types of naturally occurring DCs are observed: myeloid DCs (mDCs) and plasmacytoid DCs (pDCs).^103^, ^104^

Westdorp et al. investigated the immunological response and clinical outcomes of vaccinating chemo‐naive castration‐resistant prostate cancer patients with blood‐derived CD1c+ mDCs and pDCs (NCT02692976, Table 2). These DCs were stimulated with protamine/mRNA and loaded with TAAs, including NY‐ESO‐1. The study found that the DC vaccines were well tolerated, inducing functional NY‐ESO‐1‐specific T cells in almost half of the patients. Notably, the presence of these T cells correlated with improved clinical outcomes, particularly with a median duration of approximately 1.6 years.^105^

Ongoing efforts are directed towards enhancing the manufacturing and administration processes of DC vaccines. Currently, state‐of‐the‐art DC‐based vaccines loaded with multiple peptides, including NY‐ESO‐1, are being evaluated in different phases of clinical trials (NCT03970746, Table 2).

## 6 ARTIFICIAL ADJUVANT VECTOR CELL‐BASED VACCINE

In the evolving landscape of immunotherapy, a novel approach (aAVCs) has emerged, demonstrating the potential to elicit multifaceted immune responses. These aAVCs are engineered to carry α‐galactosylceramide (α‐GalCer), an exogenous glycolipid ligand, enabling them to perform multiple functions. Once α‐GalCer is presented on CD1d molecules, it activates invariant natural killer T (iNKT) cells, empowering them to eliminate aAVCs and release TAAs. Subsequently, DCs present these antigens, leading to the generation of CD4+ and CD8+ T cells specialised to target antitumour antigens (Figure 3).

Fujii, Shin‐Ichiro et al. developed an aAVC targeting NY‐ESO‐1 (aAVC‐NY‐ESO‐1). This innovative vaccine effectively stimulated a specific CTL response against NY‐ESO‐1, while also activating iNKT and natural killer (NK) cells. Additionally, it promoted DC maturation and facilitated the cross‐presentation of NY‐ESO‐1 antigen, leading to significant antitumour effects in preclinical mouse models.^106^ A phase 1/2 study (NCT04939701) assessed the safety, and tolerability of another aAVC product targeting NY‐ESO‐ 1 called (ASP0739), administered as a single agent and in combination with pembrolizumab. The findings from these investigations are still pending publication, but these trials represent a promising prospect for expanding cancer treatment options and addressing HLA loss, potentially circumventing the necessity for HLA matching.

## 7 WHOLE‐CELL VACCINES

Whole‐cell cancer vaccines present another broader immunotherapeutic strategy by exposing the immune system to the entire range of antigens expressed by cancer cells. This extensive exposure has the potential to activate a diverse array of immune cells, including T cells, B cells and NK cells, thereby amplifying the immune response against cancer.^107^

In a preclinical study, renal cancer cells were genetically modified to express the NY‐ESO‐1 antigen and then injected into mice with NY‐ESO‐1‐negative renal tumours. This treatment led to a significant reduction in tumour size and enhanced the interaction between DCs and T cells. These results indicate that NY‐ESO‐1, even in tumours that do not naturally express it, could function as an adjuvant, effectively training both innate immune cells and T cells to recognise and target tumour epitopes with increased immunogenicity (Figure 2).^57^

NY‐ESO‐1‐based cancer vaccines exhibit considerable potential for tumour treatment. With ongoing refinement and rigorous clinical evaluation, their efficacy in antitumour applications is becoming increasingly evident.

## 8 ADOPTIVE T‐CELL THERAPY

Adoptive T‐cell therapy is a cornerstone of cancer immunotherapy, harnessing the body's most powerful weapon in the fight against cancer. Tumour‐infiltrating autologous lymphocytes are harvested from the patient, cultivated in the laboratory, and are subsequently reintroduced into the patient in escalated quantities. The task of this army of T cells in the body is to survey, recognise tumour antigen on tumour cells, and then attack the cancer cell.

A case study demonstrated that infusing autologous T cells sensitised in vitro to recognise the 157−170 epitope of NY‐ESO‐1 in the context of HLA‐DP*04, led to a sustained clinical remission lasting up to 2 years. Notably, the CD4+ T‐cell clones persisted in the patient's blood for at least 3 months post‐administration, even in the absence of cytokine treatment. Additionally, this treatment elicited endogenous responses against melanoma antigens beyond NY‐ESO‐1.^108^

However, challenges in generating tumour‐reactive autologous tumour‐infiltrating lymphocytes from other cancer types have prompted researchers to investigate the use of autologous T cells genetically modified to produce effector T cells. This modification involves transduction with retroviral and lentiviral vectors encoding NY‐ESO‐1‐specific TCRs.

Robbins et al. embarked on the first clinical trial involving autologous T cells genetically engineered to target NY‐ESO‐1 in patients with metastatic melanoma and synovial cell sarcoma. Using a retroviral vector, they modified CD4+ and CD8+ T cells to express a TCR specific to the SLLMWITQC peptide of NY‐ESO‐1 for HLA‐A*0201, designated as IG4‐α95:LY, with alterations in the third complementarity determining region of the native 1G4 TCR α chain. Following non‐myeloablative chemotherapy and interleukin (IL)‐2 administration, these engineered T cells were infused into patients, resulting in promising response rates of 45% for melanoma and 67% for synovial cell sarcoma. Despite this initial success, the persistency of T cells among patients did not consistently align with treatment efficacy.^35^ Consequently, the study was expanded into a subsequent pilot phase trial (NCT00670748, Table 2) encompassing 20 melanoma and 18 with synovial cell sarcoma patients. The outcomes revealed objective clinical responses in 61% of synovial cell sarcoma patients and 55% of melanoma patients. In addition, 3‐ and 5‐year OS rates for synovial cell sarcoma were 38% and 14%, respectively, and 33% for melanoma at both intervals.^109^

In another phase I/II clinical trial (NCT03250325, Table 2) evaluating the safety and efficacy of novel NY‐ESO‐1‐specific TCR‐transduced T lymphocytes in synovial sarcoma patients, promising outcomes were reported. T cells were genetically engineered using a retroviral vector to express a TCR specific to the NY‐ESO‐1_157−165 peptide, with recognition restricted to HLA‐A0201 and HLA‐A0206. This engineered TCR featured two amino acid substitutions (G50A and A51E) in the CDR2 region of the native TCR‐β chain and included small‐interfering RNA sequences to suppress endogenous TCR expression. The study showed an objective response rate of 50.0% and a median OS of 650 days. Cytokine release syndrome occurred in 50.0% of subjects but was manageable with appropriate treatment. Importantly, no cases of immune effector cell‐associated neurotoxicity syndrome were reported. The infused NY‐ESO‐1‐specific T cells persisted post‐infusion and exhibited a polyfunctional phenotype, contributing to sustained antitumour effects.^110^, ^111^

Similarly, significant antitumour activity was observed in patients with metastatic synovial sarcoma, which was attributed to the prolonged persistence of adoptively transferred NY‐ESO‐1^c259^ T cells (referred to as NY‐ESO‐1 SPEAR T cells). In this context, autologous T cells were transduced with a lentiviral vector encoding the expression of NY‐ESO‐1 c259 (A*02:01 SLLMWITQC), an affinity‐enhanced TCR (lete‐cel). This approach led to antitumour responses, with an overall response rate of 20%−50% observed among four cohorts stratified based on levels of NY‐ESO‐1 expression and lymphodepletion regimens. Notably, 50% of cases experienced tumour shrinkage over several months. These engineered T cells exhibited persistent post‐infusion, with a majority displaying memory phenotypes and maintaining polyfunctionality without signs of exhaustion (NCT01343043, Table 2).^112^ However, Guillain–Barre syndrome (GBS) has been reported in two patients, presenting with symptoms such as numbness, weakness and gait abnormalities after receiving T‐cell therapy. The management of GBS involved intravenous immunoglobulin treatment. This study underscores the importance of vigilant monitoring for toxicities, such as GBS, in patients undergoing immunotherapy with genetically engineered T cells targeting specific antigens like NY‐ESO‐1.^113^

Moreover, in another study (NCT01352286, Table 2), NY‐ESO‐1 SPEAR T cells showed positive clinical responses in 80% of multiple myeloma patients. These cells exhibited cytotoxic activity and prolonged persistence, resulting in a median PFS of 1.5 years. Follow‐up research confirmed sustained efficacy, including migration to the bone marrow and ongoing tumour targeting in a subset of patients (Table 2).^114^, ^115^

In parallel, there is growing interest in combining adoptive cell therapies with cancer vaccines to potentially enhance therapeutic outcomes. Two ongoing phase I clinical trials (NCT01946373, NCT01697527, Table 2) are exploring the combination of adoptive T‐cell transfer with DC vaccination in melanoma and advanced malignancies, respectively.

### 8.1 NY‐ESO‐1 targeting innovations

**FIGURE 4:**
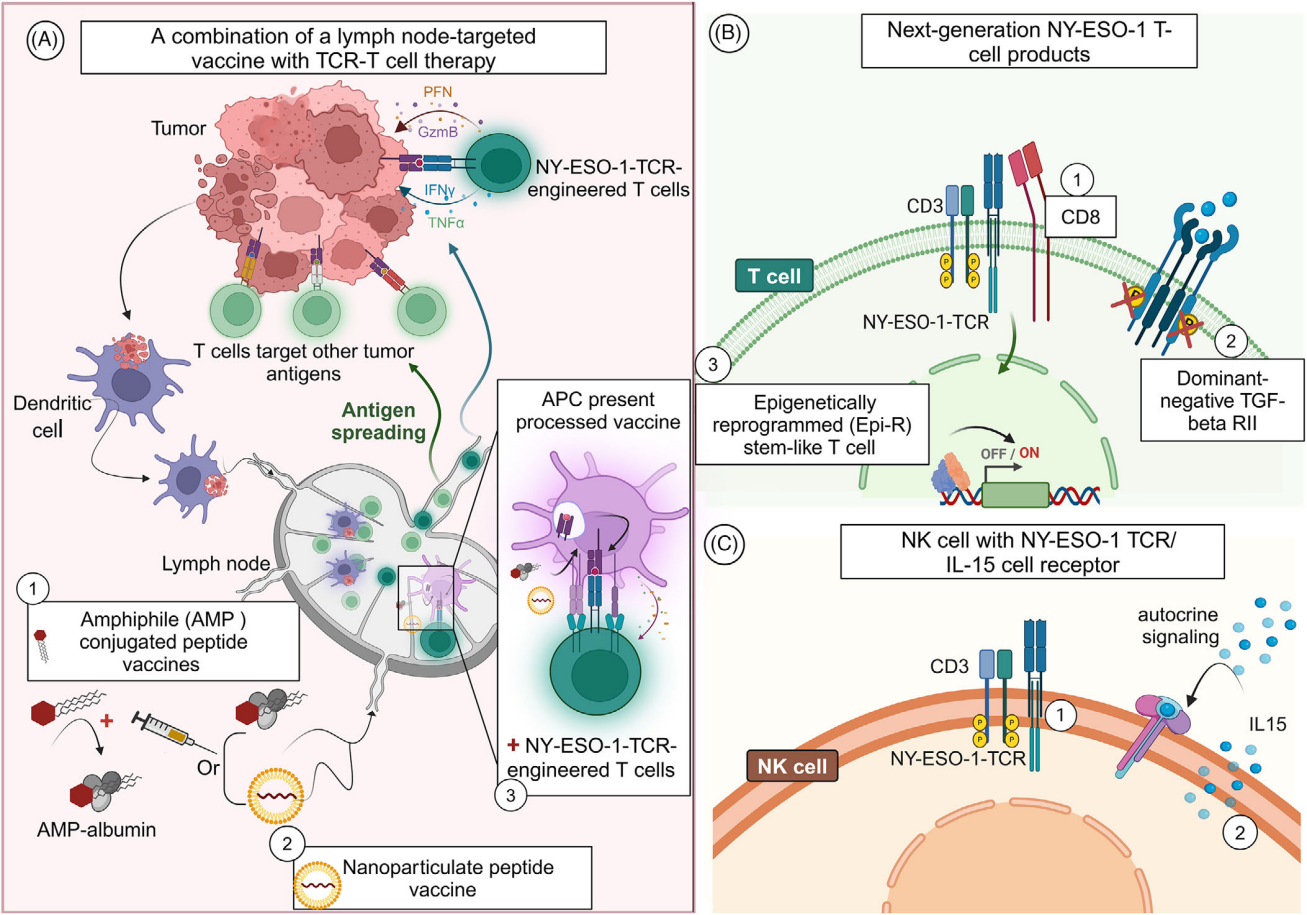
The latest tactics in New York esophageal squamous cell carcinoma (NY‐ESO‐1) targeting. (A) Integration of a lymph node‐targeted vaccine with T‐cell receptor (TCR) T‐cell therapy. Initially, Amphiphile (AMP)‐conjugated peptide vaccines bind endogenous albumin, facilitating transit to the lymph nodes (1). Alternatively, pullulan nanogels, which are immunologically inert, carry an NY‐ESO‐1 peptide and achieve selective delivery to lymph nodes through uptake by medullary macrophages, earning them the designation of an ‘immunologically stealth vaccine’ (2).^134^ Upon uptake, antigen‐presenting cells (APCs) process these vaccines to activate T‐specific endogenous T cells and adoptively transferred T cells expressing TCR specific to NY‐ESO‐1 cells (3). Activated TCR T cells migrate to tumour sites, releasing tumour‐associated antigens. Concurrently, tumour microenvironment APCs capture antigens, presenting them to T cells in lymph nodes, activating diverse T‐cell responses, including those for non‐targeted but tumour‐associated antigens. This strategy enhances immunotherapeutic efficacy, overcoming tumour immune evasion mechanisms, such as mutational antigen loss or downregulation, via antigen‐spreading through vaccination. (B) Three novel next‐generation NY‐ESO‐1 T‐cell products. These products are designed to either enhance T‐cell antigen binding and cancer cell recognition by co‐expressing the cluster of differentiation 8 (CD8) alpha receptor, modulate the tumour microenvironment by co‐expressing the dominant‐negative TGF‐beta receptor type II (dnTGF‐beta RII), or boost the stem‐like qualities of T cells through epigenetic reprogramming (Epi‐R), thereby improving their long‐term effectiveness against cancer. (C) Engineering cord blood‐derived natural killer (NK) cells with the NY‐ESO‐1 TCR/IL‐15 cell receptor, expressing CD3 and TCR signalling complexes specific to the NY‐ESO‐1 antigen, and producing interleukin (IL)‐15 for the development and maintenance of NK cells.

Recent advancements in TCR T‐cell therapy have highlighted its potential to generate significant antitumour responses. Nevertheless, obstacles persist as tumour cells frequently evade immune surveillance by diminishing or suppressing antigens, which promotes tumour progression.^116^ To overcome these challenges, researchers are developing innovative strategies to improve the efficacy of TCR T‐cell therapy. One such approach involves integrating the concept of antigen spreading, a principle that has proven effective in chimeric antigen receptor (CAR) T‐cell therapy.^117^ By combining TCR T‐cell therapy with a lymph node‐targeted vaccine, a lymphatic environment conducive to antigen spreading can be established.^118^, ^119^, ^120^, ^121^ This combination has demonstrated promise in improving durable responses against murine solid tumours resistant to TCR T‐cell monotherapy and enhancing the immune system's recognition of tumour‐specific targets beyond the original therapeutic focus, thereby fostering long‐term protection. In this study, Amphiphile (AMP)‐conjugated peptide vaccines were employed. Peptide antigens in these vaccines are coupled with lipophilic molecules that non‐covalently bind to endogenous tissue albumin at the injection site. This interaction forms AMP–albumin complexes, which regulate the entry of vaccine components into the lymph nodes. By managing this process, the complexes enhance the uptake of vaccine components by resident APCs. Consequently, this leads to the concentrated presentation of cognate peptides and costimulatory ligands, along with cytokine secretion, thereby robustly activating T cells in vivo (Figure 4A).^122^

Building on this approach, a phase I clinical trial (registration ID: JMA‐IIA00346, Table 2) combined NY‐ESO‐1‐specific TCR‐engineered T‐cell therapy with a lymph node‐targeting nanoparticulate peptide vaccine (Figure 4A). This trial involved three patients who had not undergone prior lymphodepletion and utilised TCR‐engineered T cells targeting NY‐ESO‐1, with modifications aligned with those in previous trials (NCT02366546, NCT03250325). The treatment regimen included the infusion of autologous NY‐ESO‐1‐specific TCR T cells in conjunction with a pullulan nanogel complex carrying an NY‐ESO‐1 peptide, designed to target a lectin specifically expressed by APCs, and the administration of a CpG oligoDNA adjuvant (TLR9 agonist). Notably, one patient exhibited encouraging, enduring therapeutic outcomes, including tumour shrinkage that persisted for over 2 years and the sustained presence of TCR T cells.^123^, ^124^

In addition, cancer cells evade immune surveillance through a variety of mechanisms, such as manipulation immune checkpoint molecules or the secretion of transforming growth factor β (TGF‐β), which leads to the creation of an immunosuppressive microenvironment environment.^125^, ^126^, ^127^ To counteract TGF‐β signalling in TCR‐engineered T cells, researchers have developed next‐generation NY‐ESO‐1 T‐cell products. In this advanced approach, autologous tumour‐infiltrating lymphocytes are genetically engineered to express both dnTGF‐βRII (dominant‐negative TGFΒRII)—a truncated form lacking the intracellular domain necessary for downstream signalling—and NY‐ESO‐1 TCR.^128^ The safety and efficacy of this therapeutic intervention are currently being evaluated in an ongoing clinical trial (NCT02650986, Table 2).

Furthermore, a phase II master protocol l is exploring three distinct next‐generation NY‐ESO‐1 T‐cell products. These products either co‐express the cluster of differentiation 8 (CD8) alpha cell surface receptor (GSK3901961), co‐express the dominant‐negative TGF‐beta receptor type II (dnTGF‐beta RII) cell surface receptor (GSK3845097), or are engineered using the epigenetically reprogrammed (Epi‐R) manufacturing process (GSK4427296) (Figure 4B). These approaches aim to enhance T‐cell antigen binding,^129^, ^130^ modulate the tumour microenvironment,^131^ and improve the stem‐like qualities of the T cells utilised for treatment.^132^, ^133^ This trial was terminated due to a change in GSK's R&D priorities (NCT04526509).

Amid these efforts, significant challenges remain, particularly in establishing a reliable supply of antitumour T cells and developing allogeneic ‘universal’ cell therapy products that are storable and readily available. To address this, researchers are exploring the modification of patients' own haematopoietic stem cells to function as a ‘living drug’, offering sustained immunotherapy. This approach is currently being evaluated in a phase I clinical trial, which aims to assess the efficacy of combination NY‐ESO‐1 TCR engineered haematopoietic stem cells with NY‐ESO‐1TCR‐engineered T cells across a range of cancer types (NCT03691376, Table 2).

Meanwhile, other researchers are investigating NK cells as an alternative to T cells due to their inherent ability to recognise and eliminate tumour cells, potentially bypassing concerns such as graft‐versus‐host disease.^135^, ^136^ This includes the engineering of cord blood‐derived NK cells to express the NY‐ESO‐1 TCR/IL‐15 cell receptor, incorporating the CD3 and TCR signalling complex to specifically target the NY‐ESO‐1 antigen. Additionally, these NK cells are engineered to ectopically produce IL‐15, a pleiotropic cytokine critical for the development and maintenance of NK cells (Figure 4C).^137^ The efficacy of this approach in treating solid tumours is currently being evaluated in phase I/II clinical trials (NCT06066359, Table 2).

Overall, the clinical trials of NY‐ESO‐1‐based therapies have contributed substantial insights. Table 3 provides a comprehensive overview of these strategies, emphasising their advantages, challenges and commonly used protocols.

**TABLE 3:** Therapeutic approaches targeting New York esophageal squamous cell carcinoma (NY‐ESO‐1): advantages, challenges and strategies.

	Advantages	Challenges	Stratiges
Peptide/protein vaccine	Induce humoural, cellular immunity or both, depending on the epitope or whole protein used Easy to produce and store Low production costs Chemical stability	Short half‐life TCR repertoire may not accurately reflect the naturally processed antigen Safety concerns related to the use of adjuvants and administration routes	Use of adjuvants as antigen carriers, such as liposomes or immune‐stimulating complexes (e.g., Montanide, poly‐ICLC, incomplete Freund's adjuvant, ISCOMATRIX, cholesteryl pullulan, MIS416, CHP, and monophosphoryl lipid A Infusion protein NY‐ESO‐1 with a human monoclonal antibody targeting the dendritic cell receptor DEC‐205 Combination with multiple peptides or other strategies
DNA vaccine	Can encode full‐length tumour antigens, enabling the presentation of multiple epitopes for broader T cell and humoural responses Can target multipe antigens Stable and easy to produce Low production costs	Delivery methods Potential for integration into host genome Immune tolerance	Use of vectors (e.g., plasmids) as carriers for antigens Incorporation of regulatory elements to control expression Modification of human IgG DNA to tailor antibodies, enhancing robust antitumour CD8+ T‐cell responses with high avidity Combination with other strategies and adjuvants
RNA vaccine	Similar to DNA vaccines, but does not integrate into the genome sequence	Delivery methods Stability Immune tolerance	Use of lipid/protamine nanoparticles to encapsulate mRNA, protecting it from degradation, and facilitating cell entry Combination with other strategies and adjuvants
Microbial vaccine	Act as intrinsic immune stimulants Generates potent long‐term immunity Genetically modifiable to express multiple tumour antigens Large‐scale production Cost‐effective	Safety concerns, including risks of adverse effects such as inflammation and allergic reactions Limited to certain types of pathogens Pre‐existing immunity issues	Genetic modification of bacteria to reduce virulence Engineering of viral vectors to be replication defective Utilisation of prime‐boost regimens to overcome pre‐existing immunity challenges Integration of transgenes encoding various costimulatory molecules (e.g., TRICOM, B7‐1, ICAM‐1, LFA‐3) to enhance immune response efficacy
T‐cell‐based vaccine	Effectively targets and kills tumour cells via TCR	Restricted by HLA compatibility Requires multi‐week production time for engineering genetically T cells Limited in for widespread clinical use (an autologous product) Allogeneic T cells (even if HLA‐matched) carry a risk of GVHD mediated through their native αβ T‐cell receptor High cost of therapy	Optimisation of HLA matching Genetic modification of T cells to express specific TCRs targeting tumour antigens Development of next‐generation T‐cell products aimed at modulating the tumour microenvironment and enhancing stem‐like qualities Integration with other immunotherapies and chemotherapy regimens to synergise therapeutic effects
NK cell‐based vaccine	Retain their full array of native receptors, allowing them to exert potent cytotoxic effects NK cells from cord blood can be utilised as off‐the‐shelf product for immediate clinical use (allogenic product) NK cells do not cause GVHD	Limited persistence in the body in the absence of cytokine support High cost of therapy	NK cells can be genetically modified to express TCR NY‐ESO‐1 to redirect their specificity Can be modified to ectopically produce cytokine, supporting their survival and proliferation
DC‐based vaccine	Most potent APCs Activates adaptive immune responses	Complex manufacturing process involving cell population sourcing, culture conditions, maturation cocktails and antigen loading techniques Migration to the lymph nodes	Monocytes are isolated and differentiated into immature DCs using GM‐CSF and IL‐4 DCs are loaded with tumour‐associated antigens such as peptides, nucleic acids, or tumour lysate Antigen‐loaded DCs mature using a cytokine mixture, including TNF‐α, IL‐1β, IL‐6 and prostaglandin E2^138^ DCs can be electroporated with mRNA encoding CD70, CD40 ligand, constitutively active TLR4 (TriMix), and tumour‐associated antigens to enhance antigen presentation and immune stimulation^139^
aAVC‐based vaccine	Does not require HLA matching Activates iNKT cells Harnesses both innate cells immune responses	Production complexity requires specialised handling	aAVCs are loaded with exogenous glycolipid ligands, such as agalactosylceramide (a‐GalCer), presented on CD1d molecule Activate iNKT to allow iNKT and NK cells to kill aAVCs, releasing the tumour‐associated antigen Drive generation of antigen‐specific T cells via cross‐presentation by DCs Epigenetic‐targeting drugs could represent another promising option for combination with aAVC therapy

Abbreviations: aAVC, artificial adjuvant vector cell; APC, antigen‐presenting cell; CHP, cholesterol‐bearing hydrophobised Pullulan; DC, dendritic cell; GVHD, Graft‐versus‐host disease; iNKT, invariant natural killer T; LFA‐3, lymphocyte‐function antigen 3; NK, natural killer; poly‐ICLC, polyinosinic:polycytidylic acid; TCR, T‐cell receptor; TNF‐α, tumour necrosis factor.

## 9 CONCLUSION AND FUTURE PERSPECTIVES

The NY‐ESO‐1 antigen has emerged as a promising target in cancer immunotherapy, owing to its substantial immunogenicity. It has been shown to effectively activate CD4+ and CD8+ T cells, as well as B cells, leading to a robust immune response against cancer cells. Additionally, NY‐ESO‐1's role as a diagnostic marker reinforces its value as a key candidate for targeted therapeutic strategies.

DCs are pivotal in presenting NY‐ESO‐1 antigens, a key step in initiating and amplifying antitumour immunity. This function is particularly relevant in the context of NY‐ESO‐1‐based cancer vaccines, which utilise various platforms—including protein/peptide, RNA/DNA and microbial vectors—to stimulate specific immune responses against NY‐ESO‐1‐expressing tumours. The use of DC vaccines as direct adjuvants may further enhance the therapeutic potential.

Encouraging outcomes from both preclinical and clinical studies, especially in synovial cell sarcoma and melanoma, highlight the therapeutic potential of NY‐ESO‐1‐targeted approaches. Advances in understanding clinical insights, tumour microenvironment dynamics and mechanisms of immune evasion continue to drive the development of more effective therapeutic modalities. Notably, the advent of aAVCs represents an innovative approach that may induce multifaceted immune responses, thereby contributing to the advancement of cancer immunotherapy.

The ongoing development of NY‐ESO‐1‐specific TCR‐engineered cells, including CD4+ and CD8+ T cells, reflects the dynamic evolution of targeted therapies. Although research into NK cells targeting NY‐ESO‐1 is still in its early stages, these efforts hold promise for enhancing patient outcomes and expanding therapeutic options.

However, there are significant considerations related to the expression and effectiveness of NY‐ESO‐1‐targeted therapies. For instance, the clinical trial NCT02775292 observed a decrease in NY‐ESO‐1 expression following TCR transgenic adoptive cell transfer combined with DC vaccination and PD‐1 blockade.^116^ This highlights the need for future research into strategies to enhance NY‐ESO‐1 expression and stability, such as employing DNMT inhibitors and proteasome inhibitors to improve antigen presentation and maintain immune recognition.^11^, ^140^, ^141^

Further exploration is warranted into overcoming the restricted expression of HLA class II molecules across different cancer types, which limits the full potential of CD4+ T‐cell responses. Promising approaches include enhancing NY‐ESO‐1 epitope presentation through targeted delivery to the macroautophagy pathway, such as using fusion proteins with autophagy molecules,^142^ and engineering CD4+ T cells with tumour‐specific HLA class I‐restricted TCRs.^143^ Additionally, exploring lymph node‐targeted delivery strategies may optimise therapeutic efficacy.

Monoclonal antibodies targeting immune checkpoints, such as anti‐PD‐1 and anti‐CTLA‐4, have generated significant interest for their role in unleashing T‐cell responses against tumour antigens. Combining these checkpoint inhibitors with NY‐ESO‐1‐targeted therapies holds potential for further improving treatment outcomes.

The development of TCR‐like fully human IgG1 antibodies targeting NY‐ESO‐1, along with corresponding CAR T cells, represents a cutting‐edge platform poised to advance novel therapeutics for cancer immunotherapy.^144^

In conclusion, the evolving understanding of cancer‐immune interactions, along with promising outcomes from combination therapies and ongoing research, heralds a transformative era in cancer immunotherapy. Continued efforts to overcome current challenges and explore innovative strategies will be crucial in fully realising the therapeutic potential of NY‐ESO‐1.

## AUTHOR CONTRIBUTIONS

*Visualisation, writing and creating the figures*: Alaa Alsalloum. *Reviewing and amending*: Julia A. Shevchenko. *Proofreading and supervision*: Sergey Sennikov. All authors have read and agreed to the published version of the manuscript.

## CONFLICT OF INTEREST STATEMENT

The authors declare that they have no conflicts of interest.

## ETHICS STATEMENT

Not applicable.

## CONSENT FOR PUBLICATION

All authors consent to publish.

## Data Availability

Not applicable.
